# A computational analysis of motor synergies by dynamic response decomposition

**DOI:** 10.3389/fncom.2013.00191

**Published:** 2014-01-16

**Authors:** Cristiano Alessandro, Juan Pablo Carbajal, Andrea d'Avella

**Affiliations:** ^1^AI Lab, Department of Informatics, University of ZurichZurich, Switzerland; ^2^Department of Electronics and Information Systems, Ghent UniversityGhent, Belgium; ^3^Laboratory of Neuromotor Physiology, Fondazione Santa LuciaRome, Italy

**Keywords:** muscle synergies, number of synergies, system dynamics, kinematic strokes, kinematic chain

## Abstract

Analyses of experimental data acquired from humans and other vertebrates have suggested that motor commands may emerge from the combination of a limited set of modules. While many studies have focused on physiological aspects of this modularity, in this paper we propose an investigation of its theoretical foundations. We consider the problem of controlling a planar kinematic chain, and we restrict the admissible actuations to linear combinations of a small set of torque profiles (i.e., motor synergies). This scheme is equivalent to the time-varying synergy model, and it is formalized by means of the dynamic response decomposition (DRD). DRD is a general method to generate open-loop controllers for a dynamical system to solve desired tasks, and it can also be used to synthesize effective motor synergies. We show that a control architecture based on synergies can greatly reduce the dimensionality of the control problem, while keeping a good performance level. Our results suggest that in order to realize an effective and low-dimensional controller, synergies should embed features of both the desired tasks and the system dynamics. These characteristics can be achieved by defining synergies as solutions to a representative set of task instances. The required number of synergies increases with the complexity of the desired tasks. However, a possible strategy to keep the number of synergies low is to construct solutions to complex tasks by concatenating synergy-based actuations associated to simple point-to-point movements, with a limited loss of performance. Ultimately, this work supports the feasibility of controlling a non-linear dynamical systems by linear combinations of basic actuations, and illustrates the fundamental relationship between synergies, desired tasks and system dynamics.

## 1. Introduction

Richness, flexibility, and adaptability characterize the generation of movements in many animal species. During the last century these features have fascinated many scientists, who started to investigate the possible mechanisms underlying the observed motor performance. Although many questions remain open, today there is a large consensus that motor skills may arise from a modular and hierarchical organization of the movement system (Kargo and Giszter, 2000a,b; Hart and Giszter, 2004; Ting and McKay, 2007; Bizzi et al., 2008; Kargo and Giszter, 2008; d'Avella and Pai, 2010). This idea was initially introduced by Bernstein (1967) in the context of motor redundancy, and it has then evolved into different, yet related, concepts (Flash and Hochner, 2005; Giszter et al., 2010). The common denominator of these ideas is that motor actions emerge from the combination of a limited set of modules. This strategy would reduce the number of variables to be controlled, and therefore it might simplify motor control and learning.

One of the proposed forms of modularity are the so-called muscle synergies, coordinated activations of groups of muscles (Tresch et al., 1999; Saltiel et al., 2001; d'Avella et al., 2003). Hypothetically, the central nervous system (CNS) encodes a parsimonious set of synergies and combines them in a task-dependent fashion to generate appropriate motor commands. This hypothesis is typically evaluated by analyzing the spatio-temporal regularities of electromyographic signals (EMG) recorded from a group of subjects. Decomposition-based techniques, such as principal component analysis (PCA) or non-negative matrix factorization (NMF), are used to extract the components that best reconstruct the recorded dataset. In many cases these components (i.e., synergies) appear very similar across different experimental conditions, and therefore they are regarded as an indirect evidence of the hypothesized neural modularity. This methodology has been successful in explaining muscle contractions across a wide range of complex tasks (e.g. running, walking, keeping balance, reaching and other combined movements) in humans (Ivanenko et al., 2005; Cappellini et al., 2006; d'Avella et al., 2006, 2008, 2011; Torres-Oviedo and Ting, 2007, 2010), in frogs (Giszter et al., 1993; Mussa-Ivaldi et al., 1994; Kargo and Giszter, 2000b, 2008; Mussa-Ivaldi and Bizzi, 2000), cats (Ting and Macpherson, 2005; Torres-Oviedo et al., 2006), monkeys (Overduin et al., 2008, 2012), and other species (Dominici et al., 2011). However, the results are often descriptive in nature and they do not offer a principled investigation of the hypothesized synergy-based control strategy (Alessandro et al., 2013).

The implementation of muscle synergies within the CNS is currently under investigation (Bizzi and Cheung, 2013). Recently, Hart and Giszter (2010) have provided direct evidence that dedicated sets of spinal interneurons are associated to the temporal activations of synchronous synergies in frogs. Experiments with monkeys (Overduin et al., 2012) and humans (Cheung et al., 2009b; Clark et al., 2010) suggest that synergies may be organized in the spinal cord and in the cortico-spinal divergent connectivity, and that the motor cortex modulates their recruitment. For visually guided tasks, time-varying synergies might be represented also at the cortical level; their spatial structure might derive from divergent corticospinal connectivity or from spinally organized modules, and their temporal characteristic may originate from the activation dynamics of the motor cortex (d'Avella et al., 2006, 2008, 2011).

While these studies focus on physiological aspects of the muscle synergy hypothesis, very little research addresses the theoretical foundation of the proposed modular controller. Which synergies should be employed to execute the desired motor tasks? How many synergies are needed? How does the dynamics of the system to be controlled affect the synergy-set? Is there a relation between the desired tasks and these elementary control modules? Addressing these theoretical questions would certainly provide a better understanding of the muscle synergy hypothesis, and might eventually lead to a computational model to explain the experimental data. In this paper we analyze these aspects from the perspective of controlling an idealized arm. We formulate control signals for a planar kinematic chain as linear combinations of a small set of predefines actuations (i.e., synergies) in accordance with the model of time-varying synergies (d'Avella et al., 2003). For this purpose we propose the dynamic response decomposition (DRD), a general tool to find the open-loop controllers that enable a dynamical system to solve desired tasks (Alessandro et al., 2012; Carbajal, 2012). Our method initially solves the task in state variables by interpolation; then, it identifies the combination of synergies (i.e., actuation) that leads to the closest kinematic trajectory to the computed interpolant. Additionally we propose a procedure to synthesize a limited set of effective synergies. In this manuscript we apply the DRD to point-to-point reaching tasks, and to via-point movements. Within the latter class of tasks we analyze two specific scenarios: (1) moving to a desired target and coming back to the initial posture (i.e., reversal task) and (2) reaching a desired location, passing though a given via-point (i.e., via-point reaching). Our theoretical analysis is independent of the biological implementation details of muscle synergies; i.e., we employ a kinematic chain instead of a biologically plausible musculoskeletal model, and DRD is currently not proposed as a model of the CNS mechanisms underlying muscle synergies. However, we believe that our results have a general validity as they interpret the fundamental problem of controlling a non-linear dynamical system by means of a modular synergy-based controller.

Reversal and via-point reaching movements can be subdivided in two distinct kinematic phases: from the initial to the intermediate point, and from the intermediate to the final point. A possible strategy to solve these tasks is therefore to concatenate the actuations associated to the two phases; each actuation is in turn realized as a combination of synergies. This idea is related to another form of modularity, the composition of movements into sequences of kinematic primitives, or strokes (Flash et al., 1992; Novak et al., 2003). While this segmentation explains a vast amount of experimental data, there is no consensus on whether such strokes effectively reflect a segmented control strategy (Fishbach et al., 2005, 2007). Alternatively they could just emerge as a result of a possible trajectory optimization (Dagmar and Schaal, 1999), or even be artifacts of the data analysis. In these latter cases the actuation could be computed in its entirety without concatenation. In this manuscript we analyze both strategies: the concatenation of simple synergy-based control signals, and the computation of a synergy-based actuation for the whole task. This investigation provides some computational insights on the advantages and the disadvantages of these two approaches, and it offers a proof of concept on how muscle synergies and kinematic modularity might be integrated into a unified framework.

This paper is organized as follows. In section 2 we introduce the mathematical formulation of DRD, the method that we employ throughout the paper to synthesize synergies and to compute task solutions. Section 3 presents the results obtained for reversal and via-point reaching tasks. Such results are further discussed in section 4, where we additionally summarize and speculate on important aspects of the muscle synergy hypothesis that are highlighted by DRD; finally we provide some concluding remarks.

## 2. Methods

In this section we introduce the mathematical details of the dynamic response decomposition (DRD). After some definitions, we present the core element of the method: a general procedure to compute actuations that solve generic reaching tasks (see section 2.1). Subsequently, in section 2.2, we show how DRD can be used for the synthesis of a set of synergies.

Let us consider a differential equation modeling a physical system

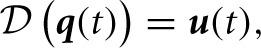



where 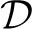 is a differential operator, ***q***(*t*) represents the time-evolution of the configuration variables (their derivatives with respect to time are $\dot{q}$(*t*)), and ***u***(*t*) is the actuation applied. Inspired by the hypothesis of muscle synergies, we formulate the actuation as a linear combination of predefined motor co-activation patterns:

(1) $$u(t)=\sum_{i=1}^{N_{\phi }}\phi_{i}(t)b_{i}:=\Phi (t)b,$$

where the *N*_ϕ_ functions ϕ_*i*_(*t*) ∈ **Φ** are called *motor synergies*, and are modulated by the weighting coefficients *b_i_*. The notation **Φ**(*t*) describes a formal matrix where each column is a different synergy, and the column vector ***b*** encapsulates the weighting coefficients. If we consider a time discretization, **Φ**(*t*) becomes a *N* dim(***q***)-by-*N*_ϕ_ matrix, where *N* is the number of time steps and dim(***q***) is the dimensionality of the configuration space. Equation (1) is essentially equivalent to the model of time-varying synergies (d'Avella et al., 2003), however, in this paper we neglect the possibility to modulate the onset time of each synergy.

We define *dynamic responses* (DR) of the set of synergies the responses **θ**_*i*_(*t*) ∈ **Θ** of the system to each synergy (i.e., forward dynamics):

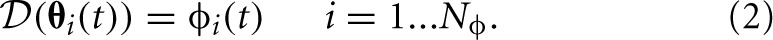



with initial conditions chosen arbitrarily.

### 2.1. The dynamic responses decomposition

A generic reaching task consists in reaching a final state (***q***_*T*_, $\dot{q}$_*T*_) from an initial state (***q***_0_, $\dot{q}$_0_) in a given amount of time *T* satisfying intermediate constraints called via-points. In the case of a single via-point defined at time *t_v_*, the task can be formalized as follows:

(3) $$\begin{array}{l} q(0)≐q_{0},\;\dot{q}(0)≐{\dot{q}}_{0},\\ q(t_{v})≐q_{v},\;\dot{q}(t_{v})≐{\dot{q}}_{v},\\ q(T)≐q_{T},\;\dot{q}(T)≐{\dot{q}}_{T}, \end{array}$$

where ≐ indicates a prescribed value, i.e., a point constraint. Depending on the desired task, more or less requirements can be imposed. For example a simple point-to-point reaching task consists only of the constraints defined at *t* = 0 and *t* = *T*. Furthermore, one could formulate via-point tasks without prescribing any velocity. This would define a class of tasks where the system is free to traverse the desired positions with any velocity. In addition, it is also possible to constrain higher order time derivatives of the configuration vector, e.g. acceleration, jerk, etc.

Controlling a system to perform a given task amounts to finding the actuation ***u***(*t*) that leads to an evolution of the system-variables that fulfills the point constraints (Equation 3). Specifically, assuming that the synergies are known, the goal is to identify the appropriate synergy combination coefficients ***b***. The DRD procedure consists of, first, solving the problem in kinematic space (i.e., finding an appropriate ***q***(*t*)), and then computing the corresponding actuation. From the kinematic point of view, solving the task can be seen as an interpolation problem; i.e., a set of functions is used to generate a trajectory ***q***(*t*) that interpolates the points {***q**_k_*(*t_k_*), $\dot{q}$_*k*_(*t_k_*)}_*k* = 0,*v*,*T*_ associated to the task-constraints (Equation 3); the idea is not to track a desired trajectory defined *a priori*, but to find any trajectory that passes through the points defined by the task. To build this interpolant one could employ orthonormal polynomials, trigonometric or Gaussian functions, to mention just a few possibilities. One of the most salient properties of DRD is that it employs the dynamic responses of the synergies (given by Equation 2), that is:

(4) $$q(t)=\sum_{i=1}^{N_{\theta }}\theta_{i}(t)a_{i}:=\Theta (t)a$$

The quality of the DRs as building blocks for the interpolation was evaluated in our previous works on planar kinematic chains (Alessandro et al., 2012) and other dynamical systems (Carbajal, 2012). As we mentioned before, if time is discretized, **Θ**(*t*) becomes a *N* dim(***q***)-by-*N*_θ_ matrix, where *N*_θ_ is the number of dynamic responses. The vector of combination coefficients ***a*** is chosen such that the task constraints are satisfied, obtaining one out of the myriad of possible trajectories that solve the task. Specifically, this vector is computed by solving the following linear system of equations:

(5) $$\left(\begin{array}{ccc} \theta_{1}(0) & \ldots & \theta_{N_{\theta }}(0)\\ \theta_{1}(t_{v}) & \ldots & \theta_{N_{\theta }}(t_{v})\\ \theta_{1}(T) & \ldots & \theta_{N_{\theta }}(T)\\ {\dot{\theta }}_{1}(0) & \ldots & {\dot{\theta }}_{N_{\theta }}(0)\\ {\dot{\theta }}_{1}(t_{v}) & \ldots & {\dot{\theta }}_{N_{\theta }}(t_{v})\\ {\dot{\theta }}_{1}(T) & \ldots & {\dot{\theta }}_{N_{\theta }}(T) \end{array}\right)a=Ma=\left(\begin{array}{c} q_{0}\\ q_{v}\\ q_{T}\\ {\dot{q}}_{0}\\ {\dot{q}}_{v}\\ {\dot{q}}_{T} \end{array}\right)=P.$$

The matrix *M* in the left-hand side is called *alternant matrix*; the solvability of the problem depends on its rank. If the matrix has full row rank, any point constraint can be solved. Otherwise, the possibility to find an exact solution (as opposed to an approximation) becomes strictly dependent on the specific task. According to the Rouché-Capelli theorem, if the rank of the alternant matrix (not necessarily equal to number of rows) is equal to the rank of the augmented matrix [*M|P*], where *P* is the vector of point constraints, the specific problem can be solved exactly. Section 3 presents some examples. These observations, and their implications for the hypothesis of muscle synergies, are further discussed in section 4.

Once a kinematic solution has been found (as a linear combination of DRs), the corresponding actuation ***ũ***(*t*) can be obtained by applying the differential operator (i.e., inverse dynamics);

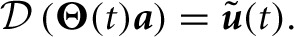



Finally, the vector ***b*** can be computed by projecting ***ũ***(*t*) onto the linear span of the synergy set **Φ**. If ***ũ***(*t*) does not belong to the linear span of **Φ**, the solution can only be approximated in terms of a defined norm (e.g. Euclidean):

(6) $$b=\underset{b}{\text{arg min}}||\tilde{u}(t)-\Phi (t)b||.$$

When time is discretized, all functions of time become vectors and this problem can be solved explicitly using the psuedo-inverse of the matrix **Φ**(*t*),

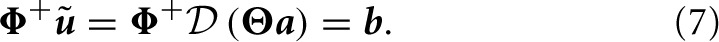



This equation highlights the mapping between the kinematic combination coefficients ***a*** (kinematic solution) and the synergy combination coefficients ***b*** (dynamic solution):

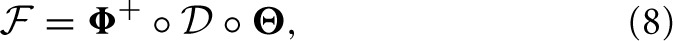



where ◦ denotes composition. Generically, this operator represents a non-linear mapping 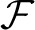:ℝ^*N*_θ_^ → ℝ^*N*_ϕ_^, and it will be discussed in section 4.3.

To assess the quality of the solution we define the following measures:

*Interpolation error*: measures the quality of the interpolant **Θ**(*t*)***a*** with respect to the task-constraints.

(9) $$\begin{array}{l} \;{\text{err}}_{I}=\sqrt{\sum_{k\in K}e_{IPk}^{2}+e_{IVk}^{2}}\\ e_{IPk}=||q_{k}-\Theta (t_{k})a||\;e_{IVk}=||{\dot{q}}_{k}-\dot{\Theta }(t_{k})a||\\ \;K=\{0,v_{1},\ldots ,v_{n},T\} \end{array}$$

where ||·|| denotes the Euclidean norm, and the difference between angles are mapped to the interval (−π, π]. The subindex *k* identifies the point constraint, i.e., *k* = 0 for the initial condition, *k* = *v_i_* for the *i*-th via-point, and *k* = *T* for the final condition. In this work we consider tasks with a single or with no via-points, i.e., *K* = {0,*v,T*} and *K* = {0,*T*}, respectively (the latter case corresponding to simple point-to-point tasks). Note that err_*I*_ is not a tracking error with respect to a predefined trajectory, but a measure of the distance between **Θ**(*t*)***a*** and the points {***q***_*k*_(*t_k_*), $\dot{q}$*_k_*(*t_k_*)} defined by the tasks.

*Projection error*: measures the distance between the actuation ***ũ***(*t*), that solves the task, and the control signal obtained by the linear combination of the synergies **Φ**

(10) $${\text{err}}_{P}=\sqrt{\int_{0}^{T}||\tilde{u}(t)-\Phi (t)b|{|}^{2}\text{d}t}.$$

This error represents the loss caused by projecting the actuation ***ũ***(*t*) onto the linear span of the synergies, and is zero only when the calculated actuation is an element of this span.

*Forward dynamics error*: measures the quality of the trajectory $\tilde{q}$(*t*, ***b***), obtained by applying the actuation **Φ**(*t*)***b*** to the dynamical system (i.e., forward dynamics), with respect to the task constraints

(11) $$\begin{array}{l} \;{\text{err}}_{F}=\sqrt{\sum_{k\in K}e_{FPk}^{2}+e_{FVk}^{2}}\\ e_{FPk}=||q_{k}-\tilde{q}(t_{k},b)||\;e_{FVk}=||{\dot{q}}_{k}-\dot{\tilde{q}}(t_{k},b)||\\ \;K=\{0,v_{1},\ldots ,v_{n},T\} \end{array}$$

Similarly to the interpolation error, err_*F*_ is not a tracking error with respect to a desired trajectory, but a measure of the distance between $\tilde{q}$(*t*, ***b***) and the points defining the tasks. Replacing $\tilde{q}$, $\dot{\tilde{q}}$, ***q***_*k*_ and $\dot{q}$_*k*_ with their corresponding end-effector values provides the *forward dynamics error of the end-effector*.

Note that the quantities err_*I*_ and err_*F*_ provide a cumulative evaluation of the DRD solution with respect to all the task-constraints. Mathematically, they represent the Euclidean distance between the DRD solution and the points characterizing the task. Since these errors are defined as a sum over quantities with different units, it could be hard to interpret them from a physical point of view. To overcome this problem, we present our results in two ways. On one hand, we present them in terms of error measures above, which provide a cumulative assessment of the results simplifying the explanation. On the other hand, we report the results in terms of the quantities *e*_IPk_, *e*_IVk_, *e*_FPk_, *e_FVk_*, which represent interpolation and forward dynamics errors with respect to position and velocity constraints independently, and therefore are susceptible to a physical interpretation. These quantities will be normalized by factors that provide references to the obtained results, and that will be defined in the next sections.

### 2.2. Synthesis and development of synergies

The synthesis of synergies is carried out in two phases: exploration and reduction. The exploration phase consists in actuating the system with an extensive set of motor signals **Φ**_0_ to obtain the corresponding DRs **Θ**_0_. The reduction phase consists in solving a small set of tasks (that we call proto-tasks, and are defined as a set of point constraints) in kinematic space, and then computing the corresponding actuations. The elements of the set **Θ**_0_ are used to interpolate the proto-tasks as described in Equations (4) and (5); the obtained trajectories are taken as the elements of the reduced set **Θ**. Finally, the synergy set **Φ** is computed by applying relation (Equation 2), i.e., inverse dynamics, to these kinematic trajectories. As a result, there will be as many synergies as the number of proto-tasks (i.e., *N*_ϕ_ = *N*_θ_).

In a nutshell, the synthesized synergies are the actuations solving the proto-tasks. A legitimate question is: “how do we choose the proto-tasks?” In principle, the DRD method does not impose any restriction. However, in order to obtain satisfactory performance, synergies should be able to approximate the desired actuations. Since the control signals corresponding to similar tasks are likely to be characterized by similar features, a reasonable choice is that the proto-tasks belong to class of the desired tasks (e.g. reversal, via-point reaching). In such a case, the synthesized synergies are actuations solving instances of the desired class of tasks, and therefore they embed the characteristic features of the desired control signals. Thus, we expect that appropriate linear combinations of these synergies are able to approximate the other actuations belonging to the desired set. In general, the more similar the proto-tasks are to the tasks to be solved (in terms of Equation 3), the better the performance of the corresponding synergies. Section 3.4 provides some examples and addresses these issues in detail.

Two other aspects that directly influence the quality of the synergy-based controller are the number of proto-tasks and their particular instances. To obtain good performance in a wide variety of tasks, the constraints defining the proto-tasks should cover relevant regions of the state space. Clearly, an increasing number of (different) proto-tasks corresponds to a gradual improvement of the overall performance. However, it also systematically expands the synergy-set, thus affecting the dimensionality of the controller. In order to tackle this trade-off, we propose a procedure that parsimoniously adds a new proto-task only when and where it is needed: if the performance in a desired task is not satisfactory, we add a new proto-task in one of the regions of the state-space with the highest projection error. In other words, the new proto-task is the task with the worst approximated actuation. Note that the procedure to evaluate the projection error in the entire workspace does not involve any actual task execution nor forward dynamics integration, and therefore it is relatively light in calculation.

## 3. Results

We apply the methodology described in section 2 to a simulated planar kinematic chain modeling a human arm [see (Hollerbach and Flash, 1982) for model details]. In the exploration phase, we employ an extensive set of motor signals **Φ**_0_ to actuate the arm model and generate the corresponding dynamic responses **Θ**_0_. The nature of these signals has a marginal role and it does not affect the quality of the obtained results (Alessandro et al., 2012; Carbajal, 2012). Here we use a set of 90 low-pass filtered uniformly random signals (butterworth with cutoff frequency of 0.314 rad). We test the performance of the method on three classes of tasks: point-to-point (section 3.1), reversal (section 3.2), and via-point-reaching (section 3.3).

### 3.1. Point-to-point tasks

**Figure 1:**
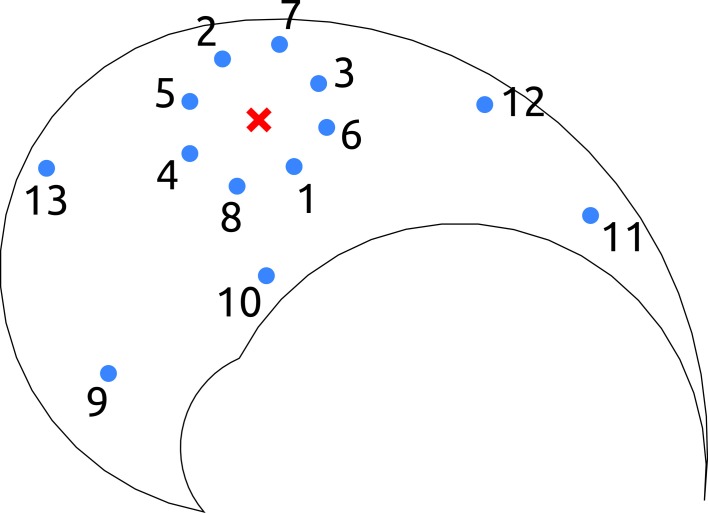
**Salient points of the testing-tasks in end-effector space**. The solid line delimits the workspace of the kinematic chain. For point-to-point testing tasks, the red cross represents the initial position of the arm, and the blue dots indicate the final targets. For reversal tasks, the red cross represents the initial and final position of the arm, and the blue dots illustrate the intermediate targets. Finally, for the via-point reaching tasks the red cross indicates the location of the via-point, and the blue dots represent the initial and the final positions of the arm. In the text, the joint configuration vector corresponding to the red cross is referred as ***q**_c_*.

A point-to-point reaching task consists in reaching a final state from an initial state in a given amount of time. Thus, a task instance is specified by four two-dimensional point constraints: initial and final joint angles and velocities. In this section we restrict our analysis to the subclass of tasks that are characterized by the initial position ***q**_c_* (red cross in Figure 1), and that impose initial and final velocities equal to zero, i.e., $\dot{q}$_*T*_ = $\dot{q}$_0_ = 0. The only unspecified constraints are the joint-coordinates of the target; i.e., since the kinematic chain has two degrees of freedom (DoF) there are two free task-parameters. Essentially the arm is required to start from the configuration ***q**_c_* and reach a desired target with zero velocity. Note that the velocity constraints are added just to restrict the class of desired tasks, and therefore to simplify the explanations throughout the paper. The method is mathematically general, and therefore can also be used to solve tasks in which these constraints are not imposed.

After the reduction phase the linear system in Equation (5) becomes:

(12) $$\left(\begin{array}{ccc} q_{c} & \ldots & q_{c}\\ \theta_{1}(T) & \ldots & \theta_{N_{\theta }}(T)\\ 0 & \ldots & 0\\ 0 & \ldots & 0 \end{array}\right)a=\left(\begin{array}{c} q_{c}\\ q_{T}\\ 0\\ 0 \end{array}\right),$$

where **θ** are the reduced DRs, and ***q**_T_* is the target posture (that uniquely defines a desired task instance as ***q**_c_* is a fixed value). Since each element is a two-dimensional column vector, the extended matrix consists of four non-zero rows; the first two rows consist of repetitions of the same numerical values (the components of ***q**_c_*). As a result, an exact kinematic solution is guaranteed if the rank of the alternant matrix is equal to 3; i.e., there should be at least three linearly independent columns. This poses a lower bound on the minimum required number of DRs and therefore of synergies. However, a higher number of synergies might be necessary to achieve satisfactory approximations of the desired actuations, and ultimately to fulfill the task requirements.

Notice that in order to obtain the alternant matrix described in Equation (13), the proto-tasks should belong to the same class of the desired tasks (i.e., point-to-point, starting at ***q**_c_*). Additionally, the exploration DRs **Θ**_0_ should be able to generate kinematic solutions that fulfills all the constraints of the proto-tasks (i.e., zero interpolation error). As it was shown by Carbajal (2012), for systems with non-linear dynamics this is likely to happen as the 8-by-90 alternant matrix, built from the exploration DRs, most probably contains more than eight linearly independent columns. Thus any point-to-point task could be solved.

**Figure 2:**
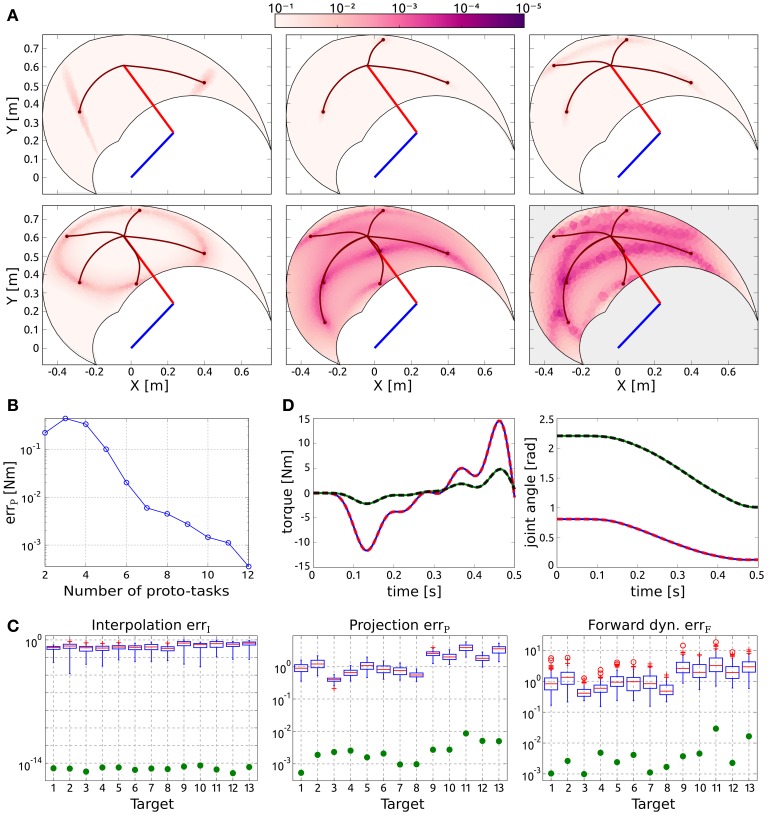
**Results of point-to-point tasks. (A)** Selection of proto-tasks based on projection error. Each panel shows the kinematic chain in its initial posture (straight segments), and the distribution of the projection error over the end-effector space (colored region). The color of each point indicates the projection error produced to reach a target in that position. The bottom right panel shows the distribution of the forward dynamics error of the end-effector using seven proto-tasks (seven synergies). **(B)** Average projection error (across targets distributed in the workspace) as a function of the number of synergies. **(C)** Evaluation of the reduction phase for the testing point-to-point tasks. Comparison between the synthesized synergies (filled circles) and subsets randomly selected from the exploration-actuations (box-plots). **(D)** Actuation that solves the task (continuous lines) and projected (dashed lines) torque, and interpolated (continuous lines) and executed (dashed lines) joint trajectories for the tasks with the highest projection error (i.e., target 11).

Figure 2A shows the distribution of the projection error for an increasing number of synergies, and exemplifies the proposed procedure to incrementally add new proto-tasks. Initially, two targets are chosen randomly (top left panel); subsequent targets are added in the regions characterized by higher projection error. As it can be seen, the introduction of new proto-tasks leads to better performance on wider regions of the space, and eventually the actuations needed to solve any point-to-point task can be reasonably approximated (err_*P*_ < 10^−2^ Nm with seven synergies). The bottom right panel shows the distribution of the forward dynamics error of the end-effector obtained with seven proto-tasks. Comparing this panel with the bottom center one (projection error with seven proto-tasks), it can be seen that the forward dynamics error reproduces the distribution of the projection error, rendering the latter a good estimate of the relative forward performance across tasks. However, it is important to stress that, due to the non-linearity of the dynamical system, the projection error serves only as an heuristic estimate of the actual error made when executing the task.

Figure 2B shows the trend of the average projection error (across the targets distributed in the workspace) as a function of the number of proto-tasks. Depending on the precision required, more or less proto-tasks can be used. Here we employ seven proto-tasks to obtain an average projection error <10^−2^ Nm. This means that the actuations to solve any point-to-point task (starting at ***q**_c_*) can be approximated by combining only seven synergies. The average forward dynamics error err_*F*_ using seven synergies amounts to ≈10^−2^. These results show that a set of “good” synergies can drastically reduce the dimensionality of the controller, while maintaining satisfactory performance. Note that the controller has to “choose” the values of two joint-torques at each time-step, thus its dimensionality is much higher than the number of DoF of the system (in fact it is infinite dimensional if we consider actuations as continuous vector-valued functions of time). Hence, seven synergies contribute a dimensionality reduction even if the system has two DoF (Alessandro et al., 2013).

To further demonstrate that the reduction phase is not trivial, we compare the errors resulting from the set of seven synthesized synergies, with the errors corresponding to 100 random subsets of size seven drawn from the exploration signals. The testing point-to-point tasks are identified by the 13 targets depicted in Figure 1. Figure 2C shows that the errors of the random subsets (box-plots) are always orders of magnitude higher than the errors of the synergies resulting from the reduction phase (filled circles). The seven reduced DRs lead to an alternant matrix with rank equal to 3, therefore any point-to-point constrain-vector of this class can be interpolated exactly. As a result, in contrast to the case of random DRs, the obtained interpolation error is negligible for all the testing tasks (err_*I*_ ≃ 10^−15^ ~ 0). In terms of projection and forward dynamics error, the reduced synergies perform about 2–3 orders of magnitude better than any random subset. Additionally, they lead to high task performance (forward dynamics errors in the range [10^−3^,10^−2^]), yet greatly reducing the dimensionality of the controller.

Figure 2D exemplifies these results for the testing tasks characterized by the highest projection error (target 11). The difference between the torque that solves the task ***ũ***(*t*) (continuous lines) and that obtained as a linear combination of synergies **Φ*b*** (dashed lines) is negligible. Similarly, there is negligible difference between the kinematic solution obtained as a linear combination of DRs (continuous lines) and the trajectory resulting from the projected actuation (dashed lines).

A more detailed evaluation of the obtained results is summarized in Table 1, which presents the normalized values of interpolation and forward dynamics errors for each task-constraint separately at the target points (i.e., *k* = *T*, see Equations 9 and 11). The errors in position (*e*_IPT_ and *e*_FPT_) are normalized to ||***e**_PM_*|| = 5.02 rad, where ***e**_PM_* is a vector containing the angular ranges of the two joints (therefore encoding the maximum position error possible); the errors in velocity (*e*_IVT_ and *e*_FVT_) are normalized to ||***e**_VM_*|| = 5.70 rad/s, where ***e**_VM_* contains the peak angular velocities of the two joints across the kinematic solutions to the 13 testing tasks. As it can be seen, the very satisfactory maximum normalized values are 3.62×10^−4^ (i.e., 0.0002 rad, task 12) for position, and 5.13×10^−3^ (0.03 rad/s, task 11) for velocity forward dynamics errors.

**Table 1:** **Normalized interpolation (int) and forward dynamics (fwd. dyn.) errors for each task-constraint of the testing point-to-point tasks**.

**Task**	**int_*T*_ (×10^−16^)**		**fwd. dyn._*T*_ (×10^−4^)**	
	**Pos**	**Vel**	**Pos**	**Vel**
1	1.77	2.91	0.23	1.80
2	0.99	3.77	1.14	4.50
3	0.99	0.75	1.34	1.27
4	4.51	0.97	0.96	8.45
5	3.78	2.45	0.22	4.20
6	0.91	1.70	0.64	7.23
7	1.59	3.66	0.48	1.91
8	1.76	2.41	0.86	2.87
9	5.59	2.02	1.13	6.47
10	4.53	6.56	1.05	7.93
11	0	2.98	3.38	51.3
12	0.88	0.25	3.62	2.38
13	2.21	5.39	1.37	28.9

*The normalization factors are ||**e**_PM_|| = 5.02 rad and ||e_VM_|| = 5.70 rad/s for position and velocity errors, respectively; the rationale behind these factors is discussed in section 3.1. The errors are evaluated at the time of the target constraint T. The expressions pos and vel identify position and velocity constraints, respectively*.

### 3.2. Reversal tasks

A reversal task consists in reaching a desired target and coming back to the initial position. The tasks considered in this subsection are characterized by zero velocity at the time of the constraints, i.e., $\dot{q}$(0) = $\dot{q}$(*t_v_*) = $\dot{q}$(*T*) = 0, and by the initial (and final) posture placed in the center of the operational space, i.e., ***q***(0) = ***q***(*T*) = ***q***_*c*_ (red cross in Figure 1). Thus, the only free task-parameters are the joint-coordinates of the intermediate target (two parameters). In other words, the agent is required to reach a certain location with zero velocity (i.e., the via-point), and return to its initial posture. These reversal tasks have relevance as they resemble the motion performed for carrying objects to and from the agent, e.g. reaching for food and bringing it to the mouth, or picking up a salient object and moving it closer for examination.

After the reduction phase, the linear system of Equation (5) becomes:

(13) $$\left(\begin{array}{ccc} q_{c} & \ldots & q_{c}\\ \theta_{1}(t_{v}) & \ldots & \theta_{N_{\theta }}(t_{v})\\ q_{c} & \ldots & q_{c}\\ 0 & \ldots & 0\\ 0 & \ldots & 0\\ 0 & \ldots & 0 \end{array}\right)a=\left(\begin{array}{c} q_{c}\\ q_{v}\\ q_{c}\\ 0\\ 0\\ 0 \end{array}\right).$$

where **θ** are the reduced DRs, and ***q**_v_* is the intermediate desired position (that uniquely defines the specific task instance). For the same rationale discussed in section 3.1, to guarantee the existence of an exact kinematic solution for any reversal task belonging to this class, the rank of the alternant matrix, and therefore the minimal number of DRs, should be equal to 3. However, the number of synergies required to obtain satisfactory values of projection and forward dynamics errors might be higher.

**Figure 3:**
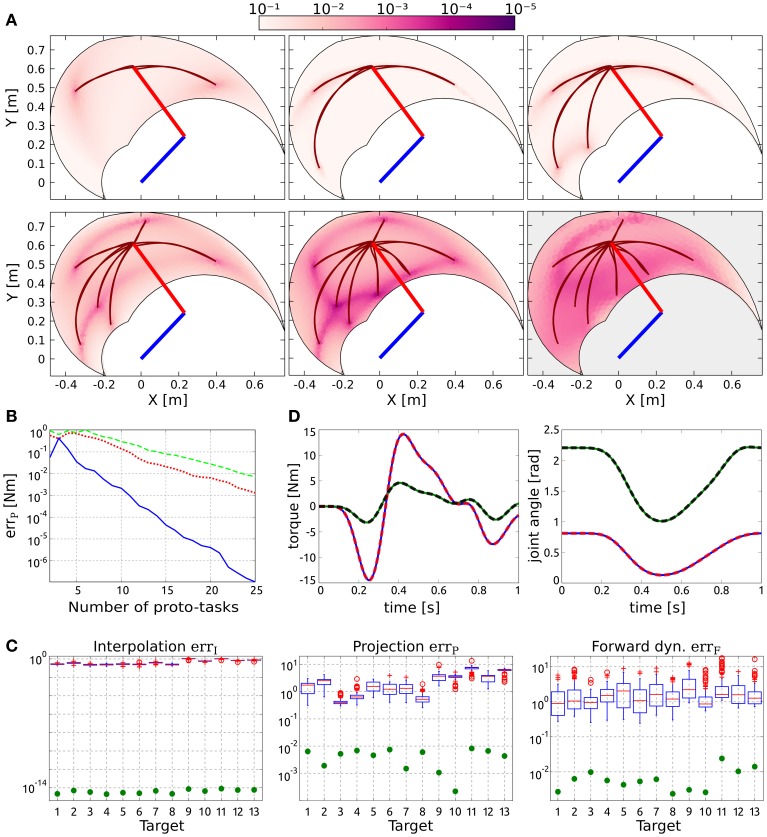
**Results of reversal tasks. (A)** Selection of proto-tasks based on projection error. Each panel shows the kinematic chain in its initial posture (straight segments), and the distribution of the projection error over the end-effector space (colored region). The color of each point indicates the projection error produced to reach that position and to go back to the initial posture. The bottom right panel shows the distribution of the forward dynamics error of the end-effector using eight proto-tasks (eight synergies). **(B)** Averaged projection error as a function of the number of proto-tasks for increasingly general classes of via-point tasks. The least general tasks are reversal motions (blue continuous line), characterized by two free task-parameters (i.e., configuration of the intermediate target). An increase in generality consists in fixing only the initial posture, while intermediate target and final position represents free task-parameters (red dotted line). Finally the most general class (green dashed line) does not fix any posture (six free task-parameters). The number of synergies required to achieve the same error increases with the generality of the class of tasks. These results are discussed in section 3.4. **(C)** Evaluation of the reduction phase for the testing reversal tasks. Comparison between the synthesized synergies (filled circles) and subsets randomly selected from the exploration-actuations (box-plots). **(D)** Actuation that solves the task (continuous lines) and projected (dashed lines) torque, and interpolated (continuous lines) and executed (dashed lines) joint trajectories for the tasks with the highest projection error (i.e., target 11).

Like in the case of point-to-point movements, proto-tasks belong to same class of the desired tasks (i.e., reversal, ***q***_0_ = ***q***_*T*_ = ***q***_*c*_), and they are added incrementally. Since the position of the desired intermediate target is the only unknown, the newly added proto-task is identified by placing the via-point in the region of the operational space with the highest projection error. As shown in Figure 3A, this strategy aims at decreasing the projection error over the entire configuration space, such that eventually the actuations necessary to solve any reversal task can be approximated satisfactorily. In particular, eight synergies are enough to obtain an average projection error err_*P*_ < 10^−2^ Nm (see Figure 3B, blue line), and an average forward dynamics error of ≈10^−2^.

The reduced synergies are compared to 100 subsets of 8 actuations, randomly chosen from the exploration motor signals. The testing reversal tasks are identified by the 13 intermediate targets depicted in Figure 1. The results shown in Figure 3C provide additional evidence that the reduction phase identify effective synergies: the mean errors of the random subsets (boxplot) are orders of magnitude higher than those corresponding to the reduced synergies (filled circles), and the forward dynamics errors lie in the range [10^−3^, 10^−2^], meaning that the 13 approximated actuations lead to good task performance. Figure 3D depicts the DRD solution of the task with highest projection error (target 11). The difference between computed and projected torques, as well as the difference between computed and executed trajectories are negligible, showing the quality of the synthesized synergies.

The values of the normalized interpolation and forward dynamics error for each task constraints are summarized in Table 2. The normalization factors, computed as in section 3.1, are ||***e**_PM_*|| = 5.02 rad, and ||***e**_VM_*|| = 8.20 rad/s, for position and velocity errors, respectively. The maximum normalized values of the errors are 1×10^−3^ (i.e., 0.005 rad, task 12, *k* = *T*) for position, and 2.5×10^−3^ (0.02 rad/s, task 11, *k* = *T*) for velocity forward dynamics errors.

**Table 2:** **Normalized interpolation (int) and forward dynamics (fwd. dyn.) errors for each task-constraint of the testing reversal tasks**.

**Task**	**int_*v*_ (×10^−16^)**		**fwd. dyn._*v*_ (×10^−4^)**		**int_*T*_ (×10^−16^)**		**fwd. dyn._*T*_ (×10^−4^)**	
	**Pos**	**Vel**	**Pos**	**Vel**	**Pos**	**Vel**	**Pos**	**Vel**
1	1.82	0.86	0.63	0.98	1.78	1.90	4.62	1.31
2	4.42	2.79	0.27	1.25	4.29	3.14	2.76	7.40
3	2.74	1.52	1.18	2.16	3.96	2.13	8.29	10.55
4	1.77	0.20	0.63	0.38	0.66	1.69	5.93	5.82
5	0.99	1.58	0.91	1.47	0.66	2.63	4.13	4.29
6	1.78	0.29	0.80	0.83	2.74	2.24	7.00	4.77
7	2.21	3.15	0.91	1.71	3.45	3.04	3.88	6.83
8	1.98	1.02	0.50	0.57	1.98	0.39	4.08	1.26
9	0.99	1.79	0.46	2.30	6.38	6.50	0.67	2.90
10	0.75	3.21	0.13	2.23	2.43	3.23	1.43	2.08
11	0.46	1.58	1.51	14.05	5.93	7.73	3.92	25.60
12	0.88	2.45	1.44	9.53	4.17	4.74	10.02	5.34
13	1.33	2.23	1.45	2.25	2.69	6.06	5.75	16.55

*The normalization factors are ||**e**_PM_|| = 5.02 rad and ||**e**_VM_|| = 8.20 rad/s for position and velocity errors, respectively. The errors are evaluated at the via-point (k = v) and at the final point k = T. The expressions pos and vel identify position and velocity constraints, respectively*.

#### 3.2.1. Concatenation of point-to-point actuations

Reversal tasks are composed by two kinematically different phases: from the initial point to the target (center-out), and from the target back to the initial position (out-center). Therefore, it should be possible to generate suitable control signals by concatenating the actuations associated to the individual point-to-point tasks. Each of these subtasks are solved by means of DRD. In the following we explore this possibility, and we compare the obtained solutions to the results of applying DRD to the entire reversal tasks.

In order to produce a meaningful solution from the concatenation, at the beginning of the out-center movement all the system variables (positions, velocities and accelerations) should match the values obtained at the end of the center-out phase. This condition can be enforced by imposing additional constraints on the acceleration of the joints. Here we prescribe zero velocity and acceleration at the end of the center-out tasks, at the beginning of the out-center, as well as at the target-point of the reversal tasks. Clearly, any other value would represent an equally suitable choice. Additionally, we assign zero velocity at the beginning and at the end of the reversal movements. Formally, the tasks are defined as follows:

Center-out

(14) $$\begin{array}{l} \;q(0)=q_{c},\;\dot{q}(0)=0,\\ q(t_{v})=q_{v},\;\dot{q}(t_{v})=0,\ddot{q}(t_{v})=0 \end{array}$$

Out-center

(15) $$\begin{array}{l} q(t_{v})=q_{v},\;\dot{q}(t_{v})=0,\;\ddot{q}(t_{v})=0,\\ q(T)=q_{c},\;\dot{q}(T)=0 \end{array}$$

Reversal

(16) $$\begin{array}{l} \;q(0)=q_{c},\;\dot{q}(0)=0,\\ q(t_{v})=q_{v},\;\dot{q}(t_{v})=0,\ddot{q}(t_{v})=0,\\ q(T)=q_{c},\;\dot{q}(T)=0. \end{array}$$

**Figure 4:**
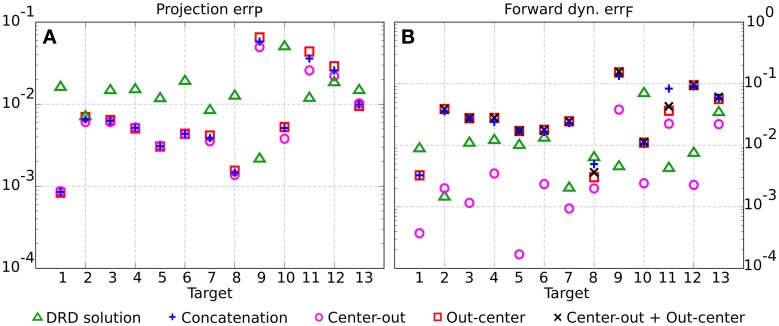
**Comparison between the DRD solutions to the entire testing reversal tasks (green triangles) and the concatenation of DRD point-to-point solutions (blue crosses) in terms of projection (A) and forward dynamics errors (B)**. The plot also indicates the performance of the individual center-out (magenta circles) and out-center tasks (red squares), and the sum of their corresponding errors (black Xs).

The synthesis of the synergies for each class of tasks follows the same procedure described in section 2.2 and exemplified in Figure 3A. We choose the number of synergies for the point-to-point (six synergies) and for the reversal tasks (seven synergies) in order to achieve comparable average projection errors across the 13 testing targets (0.011 for center-out, 0.014 for out-center, 0.016 for reversal tasks as computed by DRD, and 0.013 for the concatenation of DRD point-to-point solutions). The individual projection errors are depicted in Figure 4A. For the targets 1–8, 10, and 13, the actuations provided by the concatenation of point-to-point DRD solutions are better suited than those computed by applying DRD to the entire tasks. However, the forward dynamics errors do not always follow the same relation (Figure 4B). As an example, for the targets 2–7, the entire DRD solution performs better than the concatenation of the point-to-point actuations. The relation is, however, kept for targets 1, 8, 10, 11, and 12. Although these results might seem counter intuitive, they can be explained by analyzing the forward dynamics errors of the single center-out and the out-center tasks. It can be noticed that when the error of the entire DRD reversal solution is lower than any of the point-to-point errors, the former solution is preferable to the concatenation-based trajectory (targets 2–7, 9, 11–13). On the other hand, when the forward errors of both point-to-point tasks are lower than the error of the entire reversal solution, concatenation seems to be a better strategy (targets 1, 8, 10). In most of the cases, the forward error of the concatenation err_Fcoc_ is almost close to the “sum” of the single point-to-point errors, err_*Fco*_ and err_Foc_. In order to conform to the definition of the error (see Equation 11), this sum is computed as ${\text{err}}_{\text{Fcoc}}=\sqrt{{\text{err}}_{\text{Fco}}^{2}+{\text{err}}_{\text{Foc}}^{2}}$.

**Figure 5:**
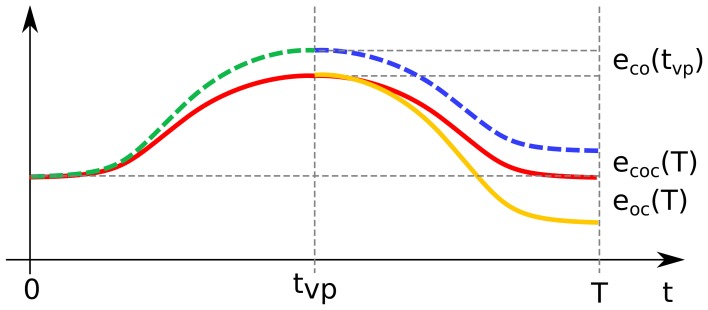
**Schematic representation of the concatenation of DRD point-to-point solutions**. The red line represents a possible exact solution to a reversal task. The first part of the concatenation-based trajectory (until the time of the via-point *t_vp_*) corresponds to the individual center-out solution (dashed green line), which is affected by the forward dynamics error *e_co_*(*t_vp_*). This error propagates over the course of the second submovement (dashed blue line), leading to the final error *e_coc_*(*T*). The latter is in general different from the final forward dynamics error *e_oc_*(*T*) of the individual out-center movement (orange continuous line).

The relation between the forward error of the concatenation and the forward errors of the individual point-to-point DRD solutions is, in reality, far from trivial. The scenario is depicted schematically in Figure 5, where the red line represents a possible solution to a reversal task. Trivially, in the first part of the movement the trajectory obtained from the concatenation strategy (dashed line) corresponds to the DRD solution to the center-out task (dashed green). The actuation corresponding to the out-center task is then applied. Since the first submotion is affected by errors (i.e., forward error of the center-out task, *e_co_*(*t_vp_*)), the system does not lie in the initial conditions associated to the out-center task (yellow line). This initial error propagates over the course of the movement according to the dynamical properties of the system (dashed blue line), and affects the state at the end of the motion. The resultant final error *e_coc_*(*T*) is in general different from the forward error of the DRD out-center solution *e_oc_*(*T*). As a result, the overall forward error of the concatenation can be higher (e.g. target 11) or lower (e.g. target 9) than the “sum” of the point-to-point errors. In theory, due to this effect, applying DRD to the entire task could lead to better performance than concatenating DRD point-to-point actuations even if the error of the entire solution is higher than both the errors of center-out and out-center tasks. Such a scenario is, however, not very likely if the error associated to center-out task is very low (as in our examples).

In general terms, none of the two methods seems to be better than the other, however, the following conclusions can be drawn. The concatenation-based solution accumulates the errors of the single movement phases. Furthermore, this strategy requires additional conditions on the kinematic variables to enable the compatibility between the two point-to-point trajectories. On the other hand, the application of DRD to the entire reversal task requires the definition of adequate proto-tasks. If these details are not available (the class of desired tasks is too general, see section 3.4), the concatenation method might be a viable alternative. Table 3, summarizes the results of this and the next sections.

**Table 3:** **Mean projection errors obtained for the testing instances of reversal and via-point reaching tasks using *N*_ϕ_ synergies**.

	**Reversal**		**Via-point reaching**	
	**Error (×10^−2^ Nm)**	***N*_ϕ_**	**Error (×10^−2^ Nm)**	***N*_ϕ_**
1st phase	1.1	6	1.2	6
2nd phase	1.4	6	1.4	6
Concatenation	1.3	12	1.5	12
DRD	1.6	7	1.3	17

*See text for more details*.

### 3.3. Via-point reaching

In this section we show the performance of DRD to solve via-point reaching tasks. These motions require the agent to reach a desired final position, passing through a given via-point. Specifically, in this section we set the via-point to be the center of the operational space ***q**_c_* (red cross in Figure 1), and the initial, intermediate, and final velocities to be equal to zero. The joint-coordinates of initial and final postures, ***q***_0_ and ***q**_T_*, represent the free task-parameters as they can be chosen arbitrarily to instantiate specific tasks (four parameters). Finally, we prescribe acceleration equal to zero at the via-point. As described in the previous section, this enables us to generate meaningful task solutions by concatenating the actuations corresponding to the two phases of the movement. Formally, the desired class of tasks can be described as follows:

(17) $$\begin{array}{l} q(0)=q_{0},\;\dot{q}(0)=0,\\ q(t_{v})=q_{c},\;\dot{q}(t_{v})=0,\;\ddot{q}(t_{v})=0\\ q(T)=q_{T},\;\dot{q}(T)=0. \end{array}$$

**Figure 6:**
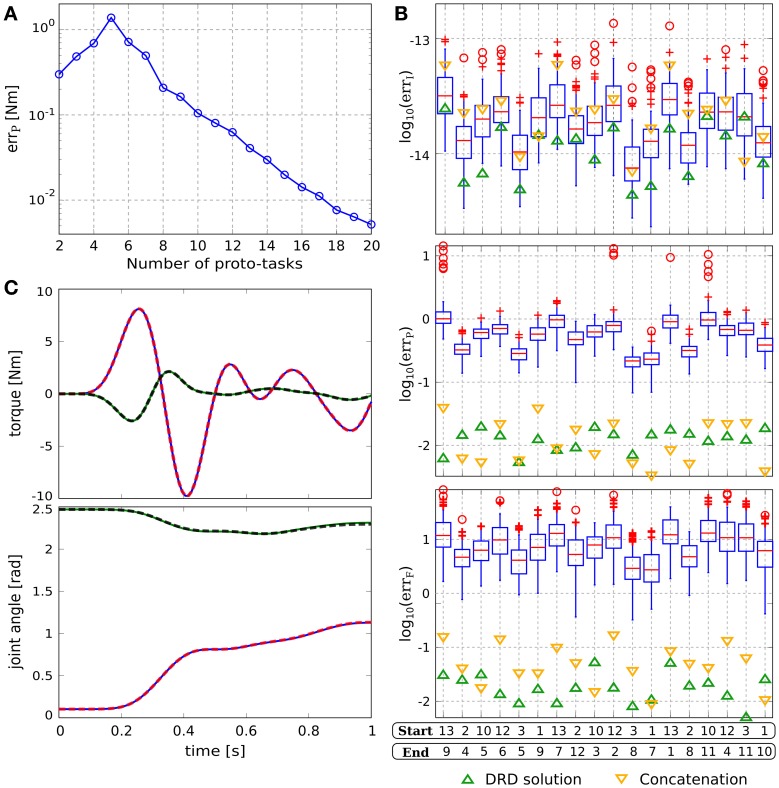
**Results of via-point reaching tasks. (A)** Average projection error (across via-point reaching tasks with initial and final positions distributed in the workspace) as a function of the number of synergies. **(B)** Evaluation of the reduction phase for 18 testing via-point reaching tasks; “Start” and “End” indicate the indexes of the initial and final points, respectively (see Figure 1). The plots also present the errors obtained by concatenating individual out-center and center-out DRD solutions (yellow downward triangles). **(C)** Actuation that solves the task (continuous lines) and projected (dashed lines) torque, and interpolated (continuous lines) and executed (dashed lines) joint trajectories for the tasks with the highest projection error (i.e., Start 10, End 5).

The synergies are synthesized as described in section 2.2. Since the parameters ***q***_0_ and ***q**_T_* can be chosen arbitrarily, the parameter space is four-dimensional. This condition does not affect the general procedure; i.e., proto-tasks are sequentially added in the point of the space characterized by the highest projection error. Figure 6A depicts the averaged projection error (across the targets distributed in the parameter space) as a function of the number of synergies.

The synthesized synergies are tested on 18 tasks, the initial and final positions of which are drawn from the targets in Figure 1. Figure 6B reports the errors obtained by using 17 reduced synergies (upward green triangles), and the performance of 100 sets of size 17 drawn from the exploration signals (box-plots). The interpolation errors corresponding to the synthesized synergies are lower than, but comparable to, the mean errors of the random sets (≈10^−14^). This is not surprising since 17 random signals are likely to produce an alternant matrix with full row-rank, thus any desired constraint vector can be obtained with negligible interpolation error. However, it is interesting to notice that the information added by the reduction phase leads to lower interpolation errors. In relation to projection and forward dynamics errors, the synthesized synergies perform about two orders of magnitude better than the random signals, providing further evidence that the reduction phase is a valuable procedure. Figure 6C shows the DRD solution of the via-point reaching task with the highest projection error (starting at point 10 and arriving at point 5). Similarly to point-to-point and reversal movements, the difference between computed and projected actuations, and the difference between interpolated and executed trajectories are negligible.

The detailed values of normalized interpolation and forward dynamics errors are summarized in Table 4. Similarly to the position and velocity errors, the acceleration errors are defined as *e_IAk_* = ||$\ddot{q}$_*k*_ − $\ddot{\Theta }$(*t_k_*)***a***|| and *e_FAk_* = ||$\ddot{q}$_*k*_ − $\ddot{\tilde{q}}$(*t_k_*, ***b***)|| (interpolation and forward dynamics, respectively). The normalization factors, computed as in section 3.1, are ||***e**_PM_*|| = 5.02 rad, and ||***e**_VM_*|| = 7.05 rad/s, for position and velocity errors, respectively; the errors in acceleration are normalized to ||***e**_AM_*|| = 61.5 rad/s^2^, where ***e**_AM_* contains the peak angular accelerations of the two joints across the kinematic solutions to the testing tasks. The maximum normalized values of the errors are 4.2 × 10^−3^ (i.e., 0.021 rad, task 10-3, *k* = *T*) for position, 6.4 × 10^−3^ (0.046 rad/s, task 13-1, *k* = *T*) for velocity, and 2.03 × 10^−6^ (1.2 × 10^−4^ rad/s^2^, task 2-8, *k* = *v*) for acceleration forward dynamics errors.

**Table 4:** **Normalized interpolation (int) and forward dynamics (fwd. dyn.) errors for each task-constraint of the testing via-point reaching tasks**.

**Task**	**int_*v*_ (×10^−16^)**			**fwd. dyn._*v*_ (×10^−4^)**			**int_*T*_ (×10^−16^)**		**fwd. dyn._*T*_ (×10^−4^)**	
	**Pos**	**Vel**	**Acc**	**Pos**	**Vel**	**Acc**	**Pos**	**Vel**	**Pos**	**Vel**
13−9	4.85	6.34	3.73	1.58	17.86	0.0127	1.59	7.90	8.19	38.26
2−4	5.16	4.12	0.27	11.79	24.88	0.0201	2.50	4.02	19.53	17.86
10−5	3.43	3.66	0.66	10.90	19.31	0.0117	1.33	5.20	35.56	28.91
12−6	3.65	0.78	2.67	9.87	12.55	0.0051	3.32	2.60	5.71	11.39
3−5	2.66	2.09	0.57	3.41	1.12	0.0001	2.50	2.91	10.93	9.66
1−9	3.19	1.42	2.17	3.41	5.05	0.0011	0.88	7.14	8.94	21.68
13−7	6.38	6.13	1.78	1.33	10.89	0.0065	1.82	3.59	4.71	5.62
2−12	5.93	4.24	1.91	7.20	11.63	0.0042	2.70	5.28	22.32	13.66
10−3	2.59	3.47	0.80	11.64	24.35	0.0189	1.11	9.02	42.07	61.72
12−2	2.83	2.88	2.58	1.70	17.67	0.0099	1.77	5.78	11.47	15.36
3−8	4.42	2.24	0.48	7.42	8.21	0.0023	1.11	1.76	3.54	4.99
1−7	0.22	3.02	0.54	0.71	7.84	0.0019	2.38	3.81	15.40	6.14
13−1	6.53	6.39	2.34	2.63	20.00	0.0190	4.53	4.86	31.77	64.69
2−8	6.25	4.53	0.47	11.59	25.12	0.0203	3.07	2.69	5.25	4.44
10−11	1.89	3.20	2.68	8.71	14.96	0.0073	0.83	17.83	18.40	22.30
12−4	2.59	0.76	2.11	10.23	11.06	0.0039	3.32	7.12	5.50	10.91
3−11	5.16	1.69	3.13	2.26	3.76	0.0005	1.25	9.98	5.42	4.22
1−10	2.50	3.77	1.01	1.93	1.25	0.0001	4.45	5.00	11.79	34.46

*The normalization factors are ||e_PM_|| = 5.02 rad, ||e_VM_|| = 7.05 rad/s and ||e_AM_|| = 61.5 rad/s^2^ for position, velocity and acceleration errors, respectively. The errors are evaluated at the via-point (k = v) and at the final point k = T. The expressions pos, vel, and acc identify position, velocity, and acceleration constraints, respectively*.

Finally, we compare the use of DRD for solving the entire tasks, to the concatenation of individual DRD point-to-point solutions. In the same vein of the reversal tasks, the considered via-point reaching movements can be composed of an initial out-center motion (from ***q***_0_ to ***q**_c_*), followed by a center-out movement (from ***q**_c_* to ***q**_T_*). The number of synergies is chosen to obtain a comparable mean projection error across the 18 testing tasks. We used six synergies for both out-center and center-out tasks, and 17 synergies for via-point reaching, leading to the following average errors: 0.012 Nm for center-out, 0.014 Nm for out-center, 0.013 Nm for via-point reaching as solved by DRD, and 0.015 Nm for the concatenation. Table 3 summarizes these results.

The yellow downward triangles in Figure 6B indicate the performance of the concatenation strategy. In line with the rationale in section 3.2.1, this method accumulates the errors of the sequential point-to-point solutions, resulting in higher values of forward dynamics and interpolation error. From the point of view of dimensionality reduction, the concatenation strategy might be convenient as the number of synergies reduces from 17 to 12 (six for each movement phase) with a small loss of performance (see Table 3).

### 3.4. Task generality and number of synergies

The obtained results show that via-point reaching tasks require a higher number of synergies than reversal tasks. To achieve a mean projection error <10^−2^ Nm, via-point reaching needs at least 17 synergies, and the reversal tasks at least 7. In this section, we provide a plausible interpretation of this difference, accompanied by additional results to support our rationale.

For the sake of clarity let us first define the *generality* of a class of tasks as the number of its free task-parameters. As discussed above, the desired class of tasks can be defined by imposing certain values to the state variables and their derivatives. For example, the reversal tasks presented in section 3.2 impose zero velocities, and additionally fix initial and final postures to a specific point of the configuration space, ***q**_c_*. Although they are essentially via-point tasks, each instance is defined only by the position of the desired intermediate target. Thus the generality of this class of task is 2 as the target is specified by two values (i.e., its joint-coordinates). Via-point reaching tasks, as defined in section 3.3, fix the position of the via-point to ***q**_c_*, and impose initial, intermediate and final velocities equal to zero; each task instance is therefore defined by the desired initial and final postures, thus the generality of this class of tasks is 4.

**Figure 7:**
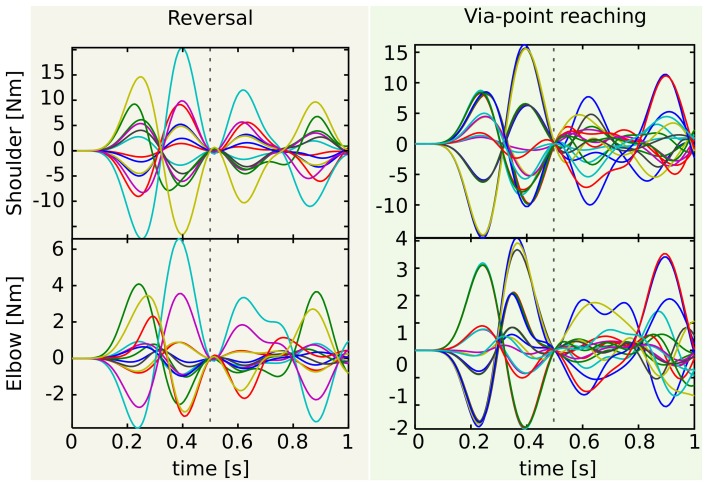
**Actuations corresponding to the testing reversal and via-point reaching tasks**. Since the via-point task is more general, the corresponding control signals **(right)** are less correlated than the reversal ones **(left)**. This is particularly visible in the second phase of the movement (after the dashed vertical line that marks the time of the via-point). See text for more details and for the values of the correlation.

The lower the generality of the desired class of tasks, the lower the variability of the control signals. This observation is exemplified in Figure 7, which shows the actuations associated to the reversal (panel **A**) and the via-point reaching testing tasks (panel **B**). As expected, the actuations in panel **A** are more regular than those in panel **B**. Quantitatively, the mean correlations between the (absolute values of the) control signals of the shoulder are 0.97 and 0.67 for reversal and via-point reaching, respectively, and the correlations between the actuations of the elbow are 0.70 and 0.53. The regularities that can be observed in the first phase of the via-point reaching movements are simply due to the fact that groups of testing tasks are characterized by the same initial position (see the abscissas label of Figure 6B). If this was not the case, the corresponding mean correlation values would be even lower.

The number of required synergies is strictly related to the previous observations. Since the proto-tasks belong to the desired class of tasks (see section 2.2), the reduced synergies are elements of the set of desired actuation. If the desired control signals are characterized by a low degree of variability (e.g. reversal case), their essential features can be captured by a handful of elements. Otherwise, a higher number of synergies is required.

To further test the validity of our rationale, we consider three increasingly more general classes of tasks. The first class (a) consists of the reversal tasks described in section 3.2, in which the only free task-parameters are the joint-coordinates of the via-point. The second one (b) fixes only the initial position, while via-point and final posture can be chosen arbitrarily. Finally, the third class of tasks (c) does not impose any fixed posture. Figure 3B shows the trends of the average projection errors as a function of the number of synergies for the three cases (blue continuous, red dotted, and green dashed lines, respectively). As expected, the number of synergies that are needed to obtain a certain degree of performance increases with the generality of the class of tasks. The projection error is meaningful only if the kinematic solution fulfills the task constraints, thus the trends in Figure 3B should be considered starting from the minimum number of proto-tasks that guarantees this condition (i.e., three, five, and six synergies). The oscillations that can be observed for a smaller number of synergies can therefore be ignored as they are not representative of task performance in any way.

**Figure 8:**
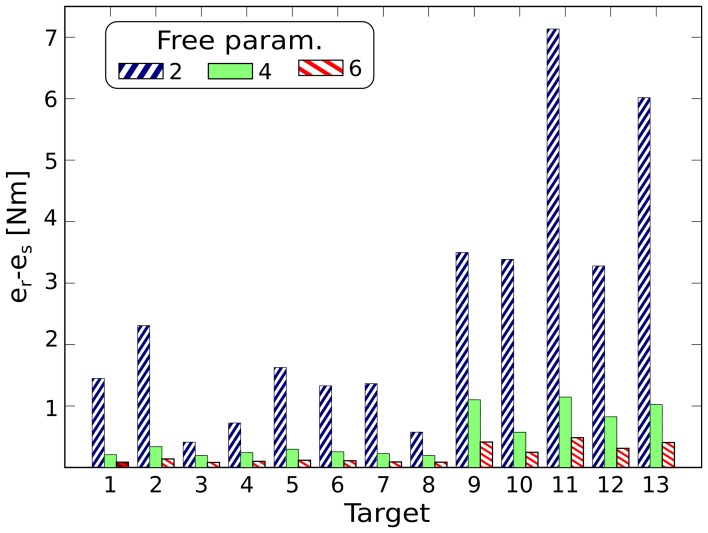
**Difference between the mean projection errors obtained by using the random sets, *e_ri_*, and the projection errors corresponding to three sets of synergies, *e_si_* (i.e., *I_i_* = *e_ri_* − *e_si_* for each set *i*), for solving the reversal testing tasks**. The sets of synergies correspond to increasingly more general classes of tasks; i.e., two, four, and six free task-parameters (right diagonal blue, green, and left diagonal red bars, respectively). This difference reduces for increasingly more general tasks, showing that the effectiveness of the reduction phase decreases as the actuations become less regular.

The effectiveness of the reduction phase is strictly related to the generality of the desired class of tasks. Very general classes lead to weakly correlated control signals. Thus, the reduction phase becomes less useful, and the synthesized synergies will embed regularities that are solely due to the dynamics of the system. Additionally, in order to obtain good performance in all the desired tasks, a large number of synergies will be required. As a direct consequence, the performance of the synthesized synergies will approach the performance of generic actuations. To illustrate this concept we compare the synergies synthesized for each of the previous classes of tasks with random sets of exploration actuations. The latter control signals are not generated through the process of reduction, and therefore they are not expected to embed any information about the tasks to be solved. We choose the minimum number of synergies that guarantees a mean projection error <10^−2^ Nm, i.e., 8, 18, and 24 for classes (a), (b), and (c), respectively (see Figure 3B). Then we use these groups of synergies to solve the 13 reversal testing tasks. Figure 8 depicts the difference between the mean projection errors obtained by using the random sets *e_ri_*, and the projection errors corresponding to the three sets of synergies *e_si_* (i.e., *I_i_* = *e_ri_* − *e_si_* for each class *i*). As expected, this difference reduces for increasingly more general tasks.

## 4. Discussion

We performed an analysis of the muscle synergy hypothesis from a computational perspective; i.e., the control of a planar kinematic chain through linear combinations of a limited set of torque profiles (motor synergies). We proposed the DRD as a tool to generate appropriate synergy-based controllers and to synthesize an effective set of synergies; such a tool has been tested to solve point-to-point and via-point tasks. DRD generates a kinematic solution by combining the dynamic responses of the synergies, and it employs inverse dynamics to compute the corresponding actuation; this control signal is finally approximated by a linear combination of synergies. The problem of finding a kinematic solution is therefore reduced to a simple interpolation, and the associated combination of synergies is obtained by projection. The quality of the obtained controller (and ultimately the task performance) depends on the set of synergies used.

Although our approach involves many assumptions and simplifications, we believe that it highlights important theoretical aspects of the muscle synergy hypothesis. First, we have provided direct evidence to the possibility of controlling a non-linear dynamical system by linear combinations of a parsimonious set of basic actuations; these scheme can result in good performance across many instances of the desired class of tasks. Hence, we support the paradigm of muscle synergies as a possible CNS principle to simplify motor control and learning. Furthermore, our results suggest that, in order to realize an effective and low-dimensional controller, synergies should embed features of the system dynamics and the desired class of tasks. Within the DRD, the information on the system dynamics is captured by the DRs (i.e., trajectories of the system variables under the actuation of each synergy), and that on the desired class of tasks is obtained by means of the reduction procedure (i.e., solving a representative set of proto-tasks). The beneficial effect of this approach is visible from two perspectives: at the kinematic level, it leads to an alternant matrix that can generate the desired constraint vectors (see Equations 12 and 13); at the actuation level, it provides samples of the desired control signals (see Figure 7). As a result, the obtained synergies over-perform hundreds of arbitrary choices of basic controllers taken from the exploration motor signals.

The number of required synergies to achieve a given performance depends on the generality of the desired class of tasks (i.e., number of free task-parameters); general tasks (e.g. via-point reaching) require more synergies than highly specific ones (e.g. reversal). These considerations further confirm that synergies are strictly tailored to the class of tasks to be solved. The mathematical formulation of DRD shows a clear non-linear relationship between kinematic and actuation modularity, that is directly intertwined to the dynamics of the system. Our analysis on the concatenation of synergy-based controllers to solve via-point tasks is directly related to the notion of kinematic primitives (Flash et al., 1992), and it represents a control scheme that, for the first time, integrates this form of modularity together with muscle synergies. The obtained results show that the concatenation method accumulates the errors of the individual submotions. On the other hand, the application of DRD to the entire via-point task requires the definition of well specified proto-tasks. If the class of task is too general, concatenation could be a viable strategy to keep the number of synergies low (see Table 3).

The usage of a kinematic chain rather than a muscle-driven skeletal model is a simplification in our work that is worth discussing. This simplification implies the definition of control signals (and therefore synergies) in the space of joint torques, and not in muscle activation space. In a musculoskeletal system, the non-linear relation between torques and kinematic variables is complemented by the additional non-linear dynamics that translates muscle activations into joint torques. The total mapping between muscle activations and kinematic variables is non-trivial. The chain of the two non-linear relations might either compensate each other, resulting in overall milder non-linearities, or form an even stronger one. A more detailed model could also bring into play other effects, for example the preflex dynamical properties of muscles, which might themselves correct mild external disturbances, stabilizing the overall system. In any case, our mathematical framework aims at capturing the fundamental theoretical problem behind the muscle synergy hypothesis; i.e., the possibility of controlling the output variables of a non-linear dynamical system (i.e., kinematic chain or musculoskeletal model) by means of a linear input strategy (i.e., linear combination of torque or muscle synergies). Thus, although muscle synergies may emerge from the interaction between neural as well as biomechanical constraints (Ting and McKay, 2007), we believe that the findings of this work (see section 4.1) are qualitatively valid also for realistic musculoskeletal models. Nevertheless, quantitative details such as the obtained number of synergies and their waveforms are strongly intertwined to the dynamical system used. We intend to evaluate DRD in more biologically plausible systems in future developments of our work. In what follows we are going to discuss our work in relation to the current debate on muscle synergies (sections 4.1 and 4.2), and to the field of robotics (section 4.3).

### 4.1. Computational insights on the muscle synergy hypothesis

Many studies in experimental neuroscience analyze the validity of the muscle synergy hypothesis solely in terms of the accuracy in approximating recorded EMG signals (d'Avella et al., 2003; d'Avella and Bizzi, 2005; Torres-Oviedo and Ting, 2007; Cheung et al., 2009a; Torres-Oviedo and Ting, 2010). This measure is equivalent to our projection error, and it does not explicitly quantifies the quality of the synergy-based controller. The introduction of complementary measures, similar to the forward dynamics error, would provide a direct evaluation of task performance, and therefore they could shed new lights on the hypothetical modularity of the CNS (Alessandro et al., 2013; Delis et al., 2013).

In this vein, some researchers introduced the concept of functional synergies, i.e., the components of an extended dataset that includes muscle activations as well as measurements of task variables (e.g. joint angles, end-limb force) (Torres-Oviedo et al., 2006; Chvatal et al., 2011). As a result, each component consists of two elements: a pattern of muscle contractions, and the corresponding evolution of the task variables. Such an approach is not too different from the idea behind DRD: synergies are associated to their DRs (i.e., biomechanical functionalities), which are linearly combined to obtain the kinematic solution of the task. However, the identification of functional synergies by means of non-negative matrix factorization (NMF), implies that muscle synergies and their biomechanical functionalities are scaled by the same coefficients. This contrasts with our theoretical results, which show a non-linear relationship (the mapping 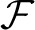, see Equation 8) between the mixing weights of the synergies and those of the DRs. Ideally, one should go beyond the use of NMF, and develop novel techniques that do not impose a linear mapping between the two sets of coefficients.

The mapping 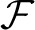 points out a fundamental non-linear relationship between kinematic and actuation modularity. More generally, this result applies to any groups of variables that are related to each other by a non-linear differential operator like 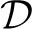 (e.g. kinematic and muscle variables, muscle excitation and activation, neural and muscle activation). However, linear forms of modularity have been investigated both at the kinematic (Berret et al., 2009) and at the muscle activation level (d'Avella et al., 2006). Our result suggests that these modularities cannot coexist; i.e., if one level of variables is bounded to a linear set (e.g. kinematic variables in our work), the other level of variables can at most be approximated linearly, but they intrinsically belong to a non-linear space (e.g. torque). Alternatively, additional processes might linearize the system dynamics as suggested by Berniker et al. (2009) and Nori (2005).

The fact that synergies and DRs are related through the dynamics of the system has another important implication. Since the former are feasible kinematic solutions to the proto-tasks, the obtained synergies can always be realized as actuations. The same cannot be said, in general, for synergies identified from numerical analyses of biomechanical data. Though some studies have verified the feasibility of the extracted synergies as actuations (Neptune et al., 2009; McGowan et al., 2010; Allen and Neptune, 2012), biomechanical constraints are not explicitly included in the extraction algorithms. Additionally, Equation (2) provides an automatic way to cope with smooth variations of the agent morphology. That is, both the synergies and their dynamic responses evolve together with the body. In line with Nori (2005), these considerations highlight the importance of the body in the hypothetical modularization of the CNS.

The mathematical formulation of DRD, and in particular the system of linear equations (5), shows a clear relation between the minimum number of synergies and the difficulty of the task. To guarantee the existence of a kinematic solution, the alternant matrix should be full-row rank. In other words, the minimum number of proto-tasks, and therefore of synergies, should correspond to the dimensionality of the task-constraint vector. For a two-DoF kinematic chain, general via-point tasks consist of three position and three velocity constraints (each of them is two-dimensional); thus, at least 12 DRs are required to be able to solve any task in kinematic space. A highly specified class of tasks reduces the minimum number of required synergies. For example, point-to-point and reversal tasks, that are characterized by two free task-parameters (i.e., location of the target), require three DRs (instead of 12); for via-point reaching this number increases to 5 (see section 3). Note that these bounds are solely based on kinematic considerations; since the dynamical system is non-linear, they do not necessarily guarantee low values of projection and forward dynamics error. In fact, as shown in section 3, the number of synergies that is required to obtain satisfactory performance is certainly higher than the theoretical kinematic-based estimation. However, this number still follows the principle that more general tasks require a higher number of synergies (see Figure 3B and section 3.4).

Our method to synthesize synergies might be interpreted from a developmental perspective. Initially, the agent explores its sensory-motor system employing a variety of actuations. Later, it attempts to solve the first tasks (proto-tasks), perhaps obtaining weak performance as the exploration phase may not have produced enough responses yet (see the box-plots in Figures 2C, 3C, and Figure 6B). If the agent finds an acceptable solution to a proto-task, such a solution is used to generate a new synergy (populating the set **Φ**), otherwise it continues with the exploration. The failure to solve important tasks for its survival, could motivate the agent to include additional proto-tasks; Figures 2A, 3A illustrate this mechanism. The development of the synergy-set incrementally improves the overall abilities of the agent. Alternatively, existing proto-tasks could be modified. It has to be clear that we are not arguing in any way that this procedure resembles the mechanisms involved in the motor development of biological organisms. It is, however, interesting that our procedure facilitates the autonomous generation of new synergies, and the possible adaptation of the existing ones to cope with the changes in the body dynamics (see Equation 2). These features are in line with the recent findings by Dominici et al. (2011). An alternative strategy for synergy development (not implemented in this paper) might be the concatenation of movement chunks. If the agent has already developed the synergies to solve point-to-point tasks, via-point proto-tasks could be solved by the concatenation of point-to-point actuations. As shown in Figures 4B, 6B the results might not be as good as if the solution were computed *ad hoc* (i.e., for the entire via-point proto-tasks). However, inspired by Sosnik et al. (2004) and Rohrer et al. (2004), one could imagine that such solutions might improve with practice, eventually leading to appropriate via-point modules.

The concatenation of point-to-point control signals to solve via-point tasks is based on the observation that movements can be composed by sequences of kinematic strokes, or submovements. The relation between this form of planning modularity (Morasso and Mussa-Ivaldi, 1982) and muscle synergies is still under debate. Possibly, as implemented in our formulation, each kinematic stroke translates into a combination of time-varying synergies, and therefore the final movement plan corresponds to a sequence of mixing patterns. This strategy would be in line with the hypothesis of an intermittent controller that sequentially initiates discrete movement primitives (Fishbach et al., 2005; Loram et al., 2010; Squeri et al., 2010; Karniel, 2013). Submovements might be combined in time succession (Soechting and Terzuolo, 1987; Meyer et al., 1988), or based on the vectorial summation of overlapping preplanned trajectories (Flash and Henis, 1991; Henis and Flash, 1995; Novak et al., 2003; Roitman et al., 2004; Pasalar et al., 2005). In this manuscript we have exemplified the former approach; the analysis of the latter by means of DRD is non-trivial, and it is therefore left for future work. As shown by recent experimental studies (d'Avella et al., 2011), such a strategy might enable reusing synergies underlying point-to-point kinematic trajectories to generate more complex trajectories involved in reaching a jumping target. Finally, it is important to notice that the kinematic solution to a via-point task appears to be composed of different movement-chunks even when it is obtained from a single composition of highly specified synergies. This observation supports the idea that strokes could just emerge as a result of the trajectory optimization (Dagmar and Schaal, 1999) or even be data analysis artifacts.

Our work analyzes the theoretical aspects, rather than the implementation details, of the muscle synergy hypothesis. As such DRD does not represent a model of the neural substrates involved in muscle synergies, and we do not claim that DRD is somehow implemented within the CNS. In fact, the biological mechanisms involved in muscle synergies are probably very different from the mathematical techniques used in this paper. For example, in our method synergies can be obtained simply by computing the solution to the proto-tasks; on the contrary, the biological process of synergy development is very likely to be incremental, and it spans several years of development (Dominici et al., 2011). However, some of the functionalities of DRD are not biologically implausible. The computation of a kinematic solution to a task (see Equation 8) can be regarded as a form of kinematic planning, and can be performed by means of a recurrent neural network (Cichocki and Unbehauen, 1992a, b) that computes the DRs mixing weights ***a***. Interestingly, DRD suggests that, although muscle synergies are defined at the motor command level (i.e., muscle activation), they could also be related to kinematic planning, and that the planning process might be carried out by exploiting knowledge of the system dynamics (in our framework embedded in the DRs). The non-linear function 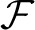 is a mapping between two finite dimensional sets of variables (the DR weights, expression of the planned trajectory, and the synergy weighting coefficients ***b***), therefore it can be encoded by means of a feedforward neural network. Conceptually, this function represents the neural pathways between the cortical areas related to planning (Buneo and Andersen, 2006) and the neural substrate where synergies are supposedly located; the outputs of this function represent the descending neural commands that modulate synergy recruitments (Ivanenko et al., 2003; Torres-Oviedo et al., 2006; Ting, 2007; Ting and McKay, 2007; Torres-Oviedo and Ting, 2010). As a matter of fact, 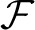 is a compact form of inverse dynamical model. Thus, its hypothetical neural implementation may involve the primary motor cortex (M1), which is know to be related to dynamical features of the movements (and in particular to inverse dynamics) (Kalaska, 2009), and the cerebellum, which is supposedly involved in the neural representation of internal models (Kawato, 1999; Diedrichsen et al., 2005; Bursztyn et al., 2006). These considerations are supported by the recent hypothesis suggesting that muscle synergies might be organized both at the spinal (Hart and Giszter, 2010) and at the cortical level (Overduin et al., 2012); their spatial structure might derive from divergent corticospinal connectivity or from spinally organized modules, and their temporal characteristic may originate from the dynamics of the recurrent connections of the motor cortex (d'Avella et al., 2006).

### 4.2. Comparison with other computational studies

While many studies try to validate or falsify the hypothesis of muscle synergies, only a few researchers have focused on developing and testing control architectures based on this concept. Some of these works aim at proposing novel techniques for robot control, other intend to analyze the hypothesized modularity from a computational point of view. Our work falls into the second category; in this section we briefly compare it to similar contributions, in particular to those studies that provide a possible interpretation of muscle synergies. The reader is referred to Alessandro et al. (2013) for a more comprehensive review.

Inspired by the original work by Mussa-Ivaldi (1997), Nori and Frezza (2005) developed a control architecture for non-linear systems based on the idea of spinal force fields (Giszter et al., 1993; Mussa-Ivaldi et al., 1994; Mussa-Ivaldi and Bizzi, 2000; Nori, 2005). Relying on the technique of feedback linearization, the method yields a set of synergies that is able to generate a complete repertoire of movements (i.e., the system can reach any arbitrary state in an arbitrary amount of time). Thus, the authors interpreted muscle synergies as a basis of the entire control action space. Berniker et al. (2009) defines synergies as the smallest set of input vectors that influences the output of a reduced-order model of the agent, and that minimally restrict the commands useful to solve the desired tasks. Practically, this set is found by optimizing the synergies against a representative dataset of desired sensory-motor signals. Similarly, Todorov and Ghahramani (2003) employ an unsupervised learning procedure to identify muscle synergies from a collection of sensory-motor data, which is obtained by actuating the robot with random signals. Their work proposes that synergies are a constituent part of an inverse model of the sensory-motor system. Another interpretation is given by Marques et al. (2012), who suggest that synergies solely reflect the biomechanical constraints of the agent.

Similar computational approaches have also been used to test whether a given model of muscle synergies (or more generally, a primitive-based controller) is competent to reproduce experimental observations. The comparison between simulated and experimental data is often performed both at the kinematic and at the muscle activation level. Furthermore, the role of biomechanical constraints is explicitly taken into account. Hence, employing biologically plausible models of the musculoskeletal apparatus becomes necessary. Kargo et al. (2010) have demonstrated that the model of premotor drives accounts for the kinematic trajectories and the isometric force fields observed in frog wiping reflexive behaviors (Kargo and Giszter, 2008). In particular they have showed that realistic wiping trajectories can be obtained simply by modulating the amplitudes and the phase-shifts of the activation pulses, without altering the muscle activation balance of each synchronous synergy. Similar studies have been carried out in the context of human walking (Neptune et al., 2009; McGowan et al., 2010; Allen and Neptune, 2012) and balancing in cats (McKay and Ting, 2008, 2012).

Unlike all those studies, the work presented herein does not aim at reproducing experimental data, rather it provides a theoretical investigation of motor synergies. As discussed in section 3.4, our work suggests that synergies can be obtained by solving well defined control problems. Similar ideas have already been proposed (Chhabra and Jacobs, 2008; Todorov, 2009; Alessandro and Nori, 2012; Thomas and Barto, 2012). However, these studies do not investigate which class of problems are best suited for this purpose. In this manuscript we show that these problems (i.e., proto-tasks) should belong to the same class of the desired tasks; this would lead to a compact set of synergies that capture features of the system dynamics and the desired class of tasks, and therefore result in good task performance. Additionally we show a clear relation between the number of synergies and two characteristics of the task: generality (i.e., number of free task parameters), and difficulty (i.e., number of constraints). Further, we propose a possible integration scheme between kinematic stroke and muscle synergies; to the best of our knowledge no other synthetic study has tested this idea.

### 4.3. The DRD method and its relevance to robotics

In robotics an active field of research focuses on novel mechanisms to generate trajectories (e.g. kinematic patterns or motor commands) and to learn their representations from given samples. The frameworks of Dynamic Movement Primitives (DMPs) (Ijspeert et al., 2013) and Stable Estimator of Dynamical Systems (SEDS) (Khansari-Zadeh and Billard, 2011) have recently received particular attention for their stability and invariance properties. Both methods encode desired trajectories in the attractor landscapes of appropriately tuned autonomous dynamical systems. While in DMPs this is obtained by modifying the dynamics of a well known system by mean of a learned forcing term, SEDS employs Gaussian mixture models (GMM) to identify the desired attractor landscape from scratch. Also the DRD can be interpreted as a method to generate kinematic trajectories and control signals. The former are obtained by linearly combining the DRs (i.e., kinematic solutions to the proto-tasks), and the latter by linearly combining the synergies (i.e., projections of the actuations that solve the proto-tasks onto the synergy-span). A quantitative comparison between our method and dynamical system-based architectures is out of the scope of this paper, however, the following considerations can be made.

DMPs and SEDS employ advanced machine-learning techniques to learn a representation of externally provided desired trajectories (e.g. via imitation learning). In contrast, DRD is not only limited to represent task solutions, but it also provides a strategy to self-generate them (i.e., planning). Given a set of constraints defining the task, DRD finds both a kinematic solution by interpolation, and the corresponding actuation by projection. As a result, no desired trajectory has to be provided externally nor any complex learning procedure is required, instead simple algebraic operations are used to solve the control problem. These features are possible mainly for two non-trivial results: (1) the dynamic responses of non-linear systems are good basis functions to build interpolant trajectories and (2) the actuations solving the proto-tasks (i.e., synergies) span a representative set of control signals.

In terms of generalization, the spatial invariance property of DMP can be exploited to generate only scaled versions of the learned movement kinematics (e.g. point-to-point reaching and reversal tasks). This is not the case for combinations of DRs, which, for example, can generate via-point reaching movements that share the same initial and intermediate points, but have different targets (see section 3.3). This kind of generalization could be obtained by shaping the dynamics of the DMPs by means of appropriate basis functions that capture common features of the desired tasks (Rückert and d'Avella, 2013). This idea is in spirit similar to solving proto-tasks, however, it requires a computationally intense learning phase if compared to our method to synthesize synergies. The same drawback is experienced by using SEDS. Furthermore, synergies embed essential features of the desired control signals, and therefore, unlike DMP and SEDS, DRD can generalize also at the actuation level.

The main disadvantage of DRD is its explicit time-indexing; as a result it does not provide an easy strategy to modulate the velocity of a given movement, and it leads to controllers that are not robust to time-perturbation. Moreover, at the current stage DRD does not provide proved stable controller, a feature that can be enjoyed in DMP and SEDS. These drawbacks could be avoided by encoding synergies and DRs by means of DMPs. In a similar vein, techniques based on mixture of DMPs have recently been proposed to improve generalization. Outstanding results have been obtained, however, each primitive has to be learned by demonstration (Muelling et al., 2010). Using DRD such primitives could be self-generated by means of the procedure to solve proto-tasks, and then they could be translated into dynamical systems. In conclusion, DRD and DMP could be combined into a unified powerful technique that inherit the advantages of both approaches, rendering the two methods more complementary than competitive.

In the DRD method, once the task is solved in kinematic space, the corresponding actuation can be computed using the explicit inverse dynamical model of the system (i.e., the differential operator 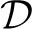). It might appear that there is no particular advantage in projecting this solution onto the linear span of the synergy set. However, the differential operator might be unknown or affected by errors; this is very often the case in robotics, where learning inverse models is still a hot topic of research (Nguyen-Tuong and Peters, 2011). A synergy-based controller would enable to compute the appropriate actuation by evaluating the mapping 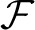 on the vector ***a***, hence obtaining the synergy combinators ***b***. Since 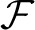 is a mapping between two finite low-dimensional vector spaces, estimating this map may turn out to be easier than estimating the differential operator 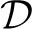. In order to estimate the map 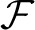, the input–output data generated during the exploration phase (i.e., **Φ**_0_ and **Θ**_0_) could be used as learning data-set. The obtained relation could be instrumental to estimate a first guess of the synergy set; 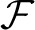 and **Φ** could then be iteratively modified until convergence. Further work is required to test these ideas.

## 5. Conclusions

The current work analyzes the hypothesis of muscle synergies from a computational perspective; i.e., the control of a planar kinematic chain through linear combinations of a limited set of torque profiles (motor synergies). The proposed DRD is able to generate effective synergies, greatly reducing the dimensionality of the problem, while keeping a good performance level. In order to obtain good performance across a variety of task instances, synergies should capture the essential features of the tasks to be solved, and take the system dynamics into account. The number of required synergies increases with the generality of the desired class of tasks. Nevertheless, to keep the number of synergies low, solutions to general tasks can be obtained by concatenating the synergy-based controllers associated to simple point-to-point movements with a limited degradation of task performance. Overall our work serves as a proof of concept for the notion of muscle synergies, showing that linear combinations of actuation modules can be used to control a non-linear dynamical system. This paper highlights the advantages and the limitations of this approach, and it draws attention to important aspects that are not easily accessible in experimental studies.

The future developments of this research point toward different directions. The relations between muscle synergies and kinematic submovements will be further investigated. In particular, we will analyze the idea of overlapping point-to-point strokes (Flash et al., 1992). Another interesting line of investigation is the validation of our method against biological data, paving the way toward a predictive model of the muscle synergy hypothesis. To this end, a first step will be the evaluation of DRD on realistic musculoskeletal models. From the theoretical point of view, we are currently studying the mathematical properties of the synergies synthesized by means of the reduction procedure. Finally, we plan to tackle the challenge of learning the mapping between kinematic and synergy coefficients.

The software used to produce all the results reported in this paper is available as a GNU Octave package under free and open source license^1^. The reader is encouraged to download, test, report bugs and submit improvements to the algorithm.

### Conflict of interest statement

The authors declare that the research was conducted in the absence of any commercial or financial relationships that could be construed as a potential conflict of interest.

## Footnotes

^1^http://users.elis.ugent.be/~jcarbaja/DRD/drd.html.
